# Primary mediastinal large B-cell lymphoma: transcriptional regulation by miR-92a through FOXP1 targeting

**DOI:** 10.18632/oncotarget.12988

**Published:** 2016-10-28

**Authors:** Martha Romero, Guillaume Gapihan, Luis Jaime Castro-Vega, André Acevedo, Li Wang, Zhao Wei Li, Morad El Bouchtaoui, Ménie Di Benedetto, Philippe Ratajczak, Jean-Paul Feugeas, Catherine Thieblemont, Carlos Saavedra, Anne Janin

**Affiliations:** ^1^ Universit**é**aris-Diderot, Sorbonne-Paris-Cit**é**Laboratoire de Pathologie, UMR-S-1165, Paris, France; ^2^ INSERM, U1165-Paris, Paris, France; ^3^ Hospital-Universitario-Fundación-Santa-Fe-de-Bogotá, Pathology-Department, Bogotá, Colombia; ^4^ INSERM, UMR970, Paris-Cardiovascular Research Center, Paris, France; ^5^ PRecherches Sino-Fran**é**s en Science du Vivant G**é**mique, Molecular-Pathology, Shanghai, China; ^6^ INSERM, U1137, Paris, France; ^7^ AP-HP-Hal Saint-Louis, Hemato-oncology-Department Paris, Paris, France; ^8^ AP-HP-Hal Saint-Louis, Pathology-DepartmentParis, Paris, France

**Keywords:** primary mediastinal large B-cell lymphoma, diffuse large B-cell lymphoma, miR-17-92 oncogenic cluster, FOXP1, bioinformatics approach

## Abstract

**Background:**

Primary mediastinal large B-cell lymphoma (PMBL) shares pathological features with diffuse large B-cell lymphoma (DLBCL), and molecular features with classical Hodgkin lymphoma (cHL). The miR-17∼92 oncogenic cluster, located at chromosome 13q31, is a region that is amplified in DLBCL.

**Methods:**

Here we compared the expression of each member of the miR-17∼92 oncogenic cluster in samples from 40 PMBL patients *versus* 20 DLBCL and 20 cHL patients, and studied the target genes linked to deregulated miRNA in PMBL.

**Results:**

We found a higher level of miR-92a in PMBL than in DLBCL, but not in cHL. A combination of *in silico* prediction and transcriptomic analyses enabled us to identify *FOXP1* as a main miR-92a target gene in PMBL, a result so far not established. This was confirmed by 3UTR, and RNA and protein expressions in transduced cell lines. *In vivo* studies using the transduced cell lines in mice enabled us to demonstrate a tumor suppressor effect of miR-92a and an oncogenic effect of FOXP1.

A higher expression of miR-92a and the down-regulation of *FOXP1* mRNA and protein expression were also found in human samples of PMBL, while miR-92a expression was low and FOXP1 was high in DLBCL.

**Conclusions:**

We concluded to a post-transcriptional regulation by miR-92a through *FOXP1* targeting in PMBL, with a clinico-pathological relevance for better characterisation of PMBL.

## INTRODUCTION

Primary mediastinal large B-cell lymphoma (PMBL), which represents 2% to 4% of all non-Hodgkin lymphoma, has been recognized as a separate entity with clinicopathological and molecular characteristics. It shares pathological features with diffuse large B-cell lymphoma (DLBCL), and molecular features and better prognosis with the nodular sclerosis subtype of classical Hodgkin lymphoma (cHL) [1]. Unlike DLBCL, which commonly occurs in older patients of both sexes, PMBL occurs predominantly in younger women, forming bulky masses with frequent invasion of adjacent structures [2]. Despite advances in treatment [3, 4], 20% of patients still succumb to their disease [5].

Gene expression profiling has enabled the identification of distinct types of DLBCL with significantly different overall survival [6]. It has also been established that the molecular signature of PMBL differs from that of other DLBCLs [7] and shares molecular features with cHL [1, 8]. Like cHL, PMBL has a distinctive cytokine signature and an activation of JAK-STAT, and NF-κB [8]. *FOXP1* (forkhead box P1), located at chromosome 3p13 [9], is an essential transcriptional regulator of B-cell development [10, 11]. FOX genes are deregulated in HL [12], and *FOXP1* is up-regulated in DLBCLs bearing the chromosomal aberrations trisomy 3 [13], t (3;14) (p13;q32) [14, 15]. *FOXP1* expression is also up-regulated in B-cell lymphoma via other mechanisms, such as B-cell activation [16] and miR-34a repression by c-Myc [17], in addition to genetic changes.

Micro RNAs (miRNAs) are a class of small, non-coding RNAs that post-transcriptionally control the translation and stability of mRNAs [18, 19]. This new class of molecules can easily be detected in fixed human tissue samples [20].

miR-17∼92 is a polycistronic miRNA cluster, with two paralogs, the miR-106a-363, and miR-106b-25 clusters [21], able to act as oncogenes [22]. It is located at chromosome 13q31 [23]. This region is amplified in lung cancer, and also in Burkitt’s lymphoma, follicular lymphoma, mantle cell lymphoma, and DLBCL [22]. The miR-17∼92 cluster has numerous biological roles [24]. In mice, its overexpression is linked to lymphoproliferative disease and autoimmunity [25] and to B-cell lymphoma [21]. In humans, an overexpression of the miR-17∼92 cluster and its paralogs has been associated with high proliferation in mantle cell lymphoma [19, 26].

The ability of miRNAs to direct the posttranscriptional repression of protein-coding genes by pairing with their mRNA enables studies on target recognition. In two genetically distinct B-cell lymphoma cell lines, miR-17∼92 transfection induced a down-regulation of different target genes: *BIM* in Raji cells, and p21 in SUDHL4 cells [27].

In AIDS-related Burkitt lymphoma and DLBCL human samples, the overexpression of miRNAs from the miR-17∼92 paralog clusters inhibited p21 [28]. In mantle cell lymphoma samples, the protein phosphatase PHLPP2, an important negative regulator of the PI3K/AKT pathway, was a direct target of miR-17∼92 miRNAs, in addition to *PTEN* and *BIM* [29].

Here we compared the expression of each member of the miR-17∼92 cluster in PMBL human samples *versus* DLBCL and *versus* cHL, and we further studied the target genes linked to deregulated miRNA in PMBL.

## RESULTS

### miR-92a was overexpressed in PMBL compared to DLBCL, but not to cHL

**Figure 1:**
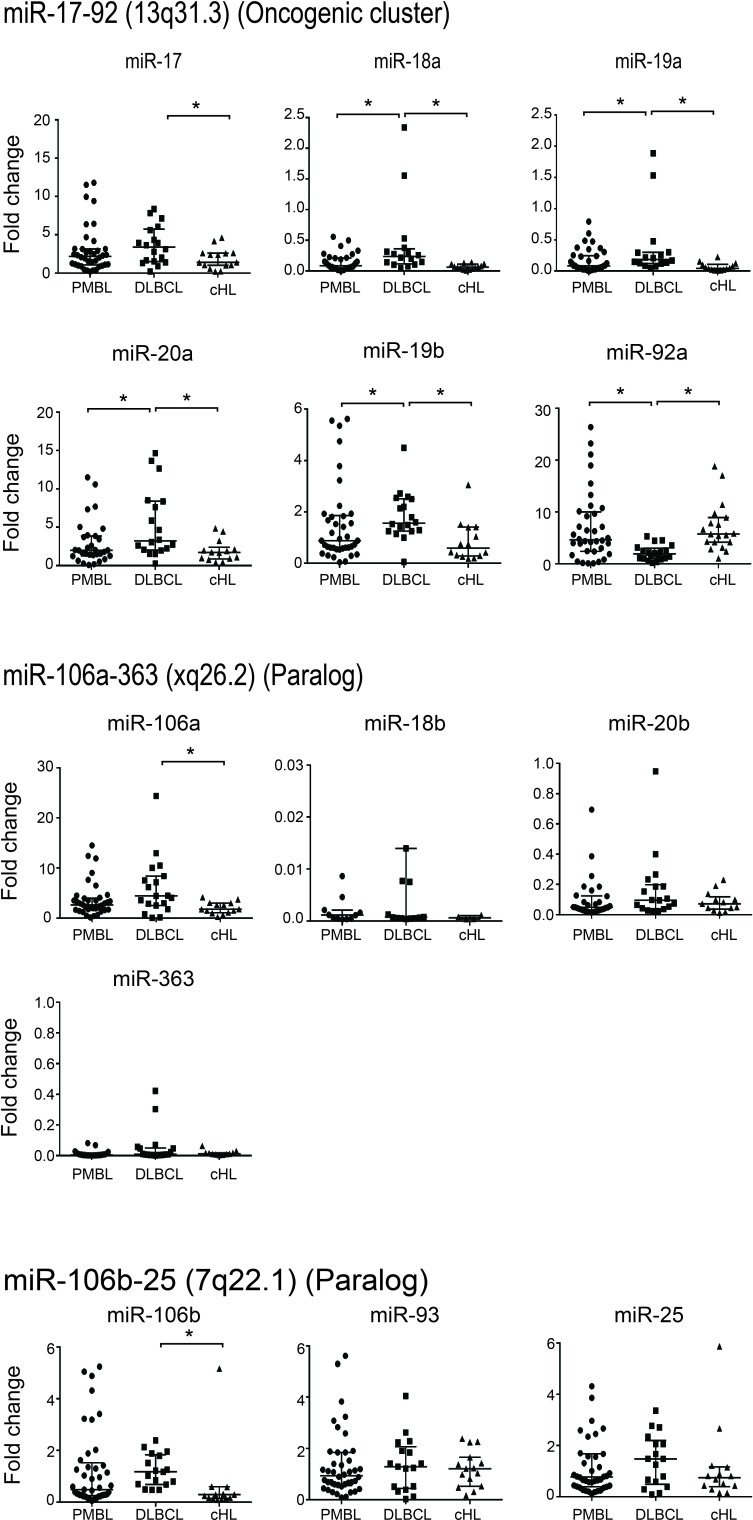
Quantification of expression levels of each microRNA in the miR-17∼92 cluster and its paralogs in 40 PMBL, 20 DLBCL and 20 cHL patient samples Results are expressed as median fold change +/- standard deviation. Statistical analyses were performed using the Mann-Whitney test. *P =< 0.001.

We quantified the expression levels of each microRNA of miR-17∼92 cluster and its paralogs in 40 PMBL, 20 DLBCL, and 20 cHL human samples (Figure 1).

When we compared PMBL and DLBCL results for the miR-17∼92 cluster, we found that only miR-92a had a significantly higher level of expression in PMBL compared to DLBCL (PMBL median 4.64 (interquartile range (Q1-Q3), 2.47-10.75); DLBCL 1.92 (Q1-Q3, 1.08-2.87); *P* =< 0.001). In contrast, miR-18a, miR-19a, miR-19b and miR-20a expression levels were significantly lower in PMBL than in DLBCL. No significant difference was found for miR-17. In the 20 DBLCL studied, there was no significant difference for miR-92a expression in GCB *versus* ABC subtypes (Supplementary Figure 2). For the two paralogs, miR-106a-363 and miR106b-25 clusters, there was no significant difference between PMBL and DLBCL.

In PMBL and cHL, we found a similar expression profile for each microRNA of miR-17∼92 cluster and its paralogs.

When DLBCL and cHL were compared, five miRNAs of the miR-17-92a cluster, but not miR-92a, and the miR-106a and miR-106b of the paralog clusters, were significantly overexpressed in DLBCL.

In the wild-type cell lines studied, we found that, as in human samples, miR-92a expression was significantly higher in Karpas than in SU-DHL-5 (Karpas 9.09 (Q1-Q3, 9-9.25); SU-DHL-5 3.3 (Q1-Q3, 3.16-3.31); *P* =< 0.002) and miR-18a significantly lower (Karpas 0.57 (Q1-Q3, 0.56-0.62); SU-DHL-5 1.17 (Q1-Q3, 0.98-1.37); *P* =< 0.02). The only discrepancy was observed for miR-20a expression which was higher in Karpas *versus* SU-DHL-5 (Karpas 17.14 (Q1-Q3, 1.9-22); SU-DHL-5 5.9 (Q1-Q3, 5.52-6.29) *P* =< 0.02), but it was significantly lower in PMBL versus DLBCL patients (Supplementary figure 3).

### miR-92a target identification in PMBL

**Figure 2:**
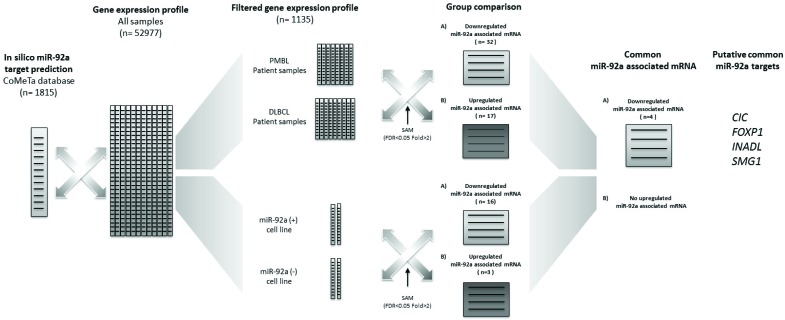
Bioinformatic approach for miR-92a target identification Analysis of the CoMeTa database, chosen to limit the number of false positive targets by a ranking method, provided a list of 1815 putative *in silico* target-genes of miR-92a. The quantile method enabled us to normalise the microarray datasets for PMBL and DLBCL patients and the four cell lines. The normalized datasets were filtered through the CoMeTa miR-92a putative target-gene list. We thus obtained a gene expression profile of 1135 filtered genes. Comparison of the filtered gene lists from PMBL and DLBCL patients enabled us to identify 32 down-regulated and 1 up-regulated putative targets of miR-92a, with a FDR<0.05 and a fold change > 2 (*q*-value < 0.05). Comparison of cell line with high expression of miR-92a (Karpas hsa-miR-92a-1 and Karpas wt) and of cell line with low expression of miR-92a (Karpas miRZip-92a and SU-DHL-5 wt) enabled us to identify 16 down-regulated and 3 up-regulated putative targets of miR-92a, with a FDR<0.05 and a fold change > 2. When patient sample and cell line results were analyzed, they had in common four down-regulated putative targets of miR-92a: *CIC*, *FOXP1, INADL*, and *SMG1*.

To identify miR-92a targets in PMBL, we combined *in silico* miR-92a target prediction and gene expression profile in PMBL and DLBCL patient samples, and in the four cell lines (Figure 2).

Analysis of the CoMeTa database, chosen to limit the number of false positive targets likely to result from a ranking method, provided a list of 1815 putative *in silico* target-genes of miR-92a. The quantile method enabled us to normalise the microarray datasets of PMBL and DLBCL patients and the four cell lines. The normalized datasets were filtered through the CoMeTa miR-92a putative target-gene list. We thus obtained a gene expression profile for 1135 filtered genes.

Comparison of the filtered gene lists from PMBL and DLBCL patients enabled us to identify 32 down-regulated and 17 up-regulated putative targets of miR-92a, with a FDR<0.05 and a fold change > 2 (*q*-value < 0.05). Comparison of cell line with high expression of miR-92a (Karpas hsa-miR-92a-1 and Karpas wt) and of cell line with low expression of miR-92a (Karpas miRZip-92a and SU-DHL-5 wt) enabled us to identify 16 down-regulated and 3 up-regulated putative targets of miR-92a, with a FDR<0.05 and a fold change > 2.

When patient samples and cell line results were analyzed, they had in common four down-regulated miR-92a associated mRNA, which were putative targets: *CIC, FOXP1, INADL*, and *SMG1* (Supplementary figures 4 and 5). There was no up-regulated miR-92a associated mRNA.

### FOXP1 as a target of miR-92a in PMBL

**Figure 3:**
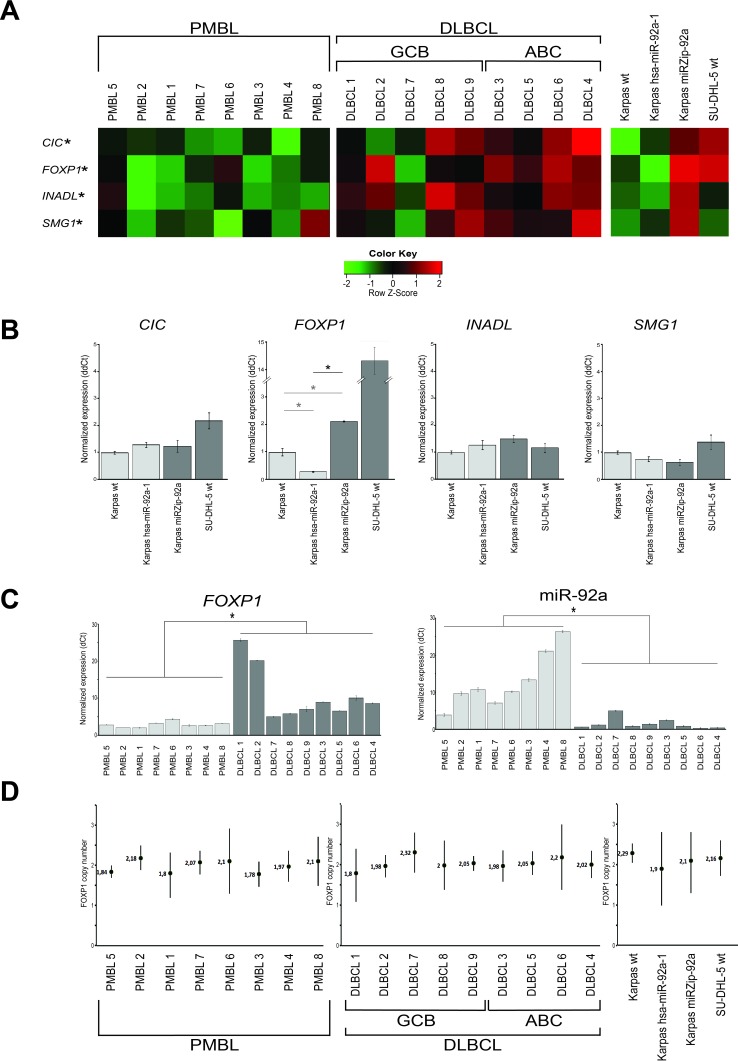
miR-92a target identification in PMBL **A**. For the 8 PMBL and 9 DLBCL patients with available cryopreserved samples, the bioinformatics approach identified 4 genes, *CIC, FOXP1, INADL* and *SMG1*, as putative targets of miR-92a, down-regulated in PMBL and cell lines overexpressing miR-92a. *q =< 0.001. **B**. The expression level of each putative target of miR-92a was assessed using Q-RT-PCR in the three Karpas cell lines. The only gene whose expression is significantly different between transduced Karpas cells underexpressing and those overexpressing miR-92a is *FOXP1* *p =< 0.001. Karpas wt *FOXP1* mRNA expression level was also significantly lower than Karpas miRZIP-92a (0.47 fold, p<0.001) and significantly higher than Karpas hsa-miR-92a-1 (3.44 fold, *p* < 0.001). **C**. When the expression levels of *FOXP1* and miR-92a were compared in PMBL and DLBCL samples, there were significantly lower levels of *FOXP1* and higher levels of miR-92a in PMBL samples. *p =< 0.001. **D**. When *FOXP1* DNA copy numbers were assessed on patient samples and cell lines using droplet digital PCR, all patient samples and cell lines studied had 2 copies of *FOXP1* gene. There was no amplification of *FOXP1*.

Transcriptomic analyses showed that the down-regulated transcripts of miR-92a associated genes *CIC, FOXP1, INADL*, and *SMG1* were highly discriminant between PMBL and DLBCL patient samples (*q*-val < 0.001)(Figure 3A).

In the two transduced cell lines, a significant difference was only found for the mRNA expression level of *FOXP1* (*p* < 0.05) (Figure 3B).

In the 8 PMBL and 9 DLBCL frozen samples, a significant difference was again only found for FOXP1 (*p* < 0.05). In addition, Kendall statistic test on Q-RT-PCR results gave a negative correlation between *FOXP1* and miR-92a expression levels (tau=-0.59; *p*-val < 0.05)(Figure 3C).

Since FOXP1 is up-regulated in DLBCL bearing the chromosomal aberration trisomy 3, we quantified the *FOXP1* DNA copy number in patient samples and cell lines. All patient samples and cell lines studied had 2 copies of *FOXP1* gene. There was no amplification of *FOXP1* (Figure 3D).

Overall these results were in favor of *FOXP1* as a target of miR-92a.

FOXP1 protein expression, studied by western-blot and immunochemistry in patient samples and transduced cell lines, confirmed the result obtained on mRNA.

**Figure 4:**
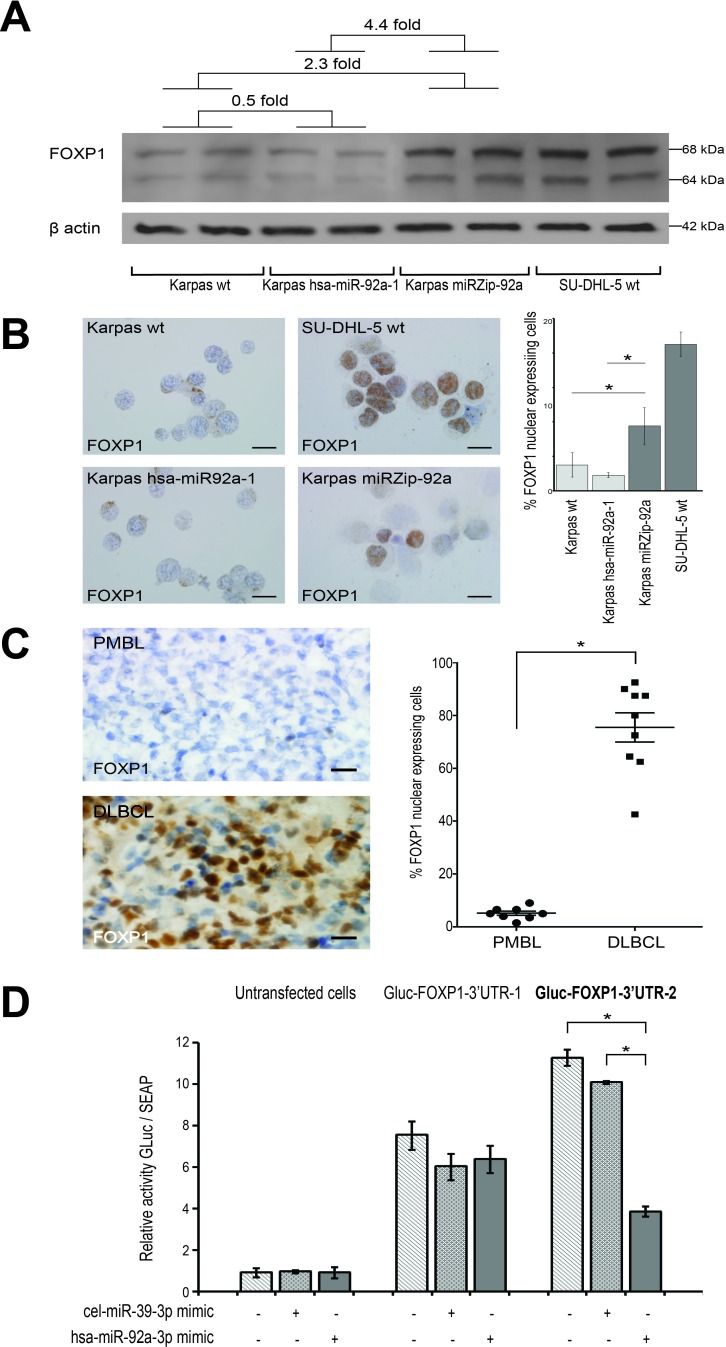
*FOXP1* as target of miR-92a in PMBL **A**. Western blot shows a FOXP1 protein expression in Karpas miRZip-92a that was 4.4 times higher than in Karpas hsa-miR-92a-1 *p =< 0.05. Karpas wt FOXP1 protein expression was also lower than Karpas miRZIP-92a (2.3 times) and higher than Karpas hsa-miR-92a-1 (0.5 times). **B**. FOXP1 nuclear expressing cells on smears from the four cell lines studied were significantly more numerous in Karpas miRZip-92a than in Karpas hsa-miR-92a-1 and Karpas wt (*p* < 0.05) (scale bar: 5μm). **C**. FOXP1 nuclear expressing cells on snap-frozen sections from PMBL and DLBCL patient samples were significantly less numerous in PMBL than in BLBCL (scale bar: 20μm). Statistical analyses were performed using the Mann-Whitney test. *P =< 0.05. **D**. Luciferase reporter activity test using 293T-cells expressing a GLuc-3′UTR_FOXP1_ fusion mRNA, transfected with a miR-92a mimic (hsa-miR-92a-3p mimic) or a negative control (cel-miR-39-3p mimic) shows a significantly lower luciferase activity in Gluc-FOXP1-3′UTR-2 transfected cells. Two plasmidic constructions were necessary to cover the whole *FOXP1* 3′UTR (Gluc-FOXP1-3′UTR-1 and Gluc-FOXP1-3′UTR-2). Results are expressed as mean luminescence activity +/- standard deviation. Statistical analyses were performed using the Mann-Whitney test. *P =< 0.05.

Western blot of FOXP1 on the four cell lines studied showed an overexpression of FOXP1 when miR-92a was down-regulated. A significantly higher expression, about 4.4 times greater of FOXP1 protein was found in Karpas miRZip-92a compared to Karpas hsa-miR-92a-1 (*p* < 0.05)(Figure 4A).

Counts of cells with nuclear FOXP1 expression in immunochemistry showed a significant difference between the two transduced cell lines (*p* < 0.05)(Figure 4B), and also between the two groups of PMBL and DLBCL patients (*p* < 0.05)(Figure 4C).

Overall, these results demonstrated a strong association between miR-92a expression level and FOXP1.

To demonstrate that *FOXP1* was a direct target of miR-92a, we performed a luciferase reporter activity test using 293T-cells expressing a GLuc-3′UTR_FOXP1_ fusion mRNA, which were transfected with a miR-92a mimic. Two plasmidic constructions were necessary to cover the whole *FOXP1* 3′UTR. When luciferase activity of these cells was assessed 72h after the miR-92a transfection, a significant reduction in luminescence was measured with the Gluc-FOXP1-3′UTR-2 construction, while no significant difference was observed with the Gluc-FOXP1-3′UTR-1 construction (Figure 4D). We used the miRNA target prediction database TargetScan to identify a 7mer-A1 miR-92a-3p binding site on the *FOXP1* 3′UTR, which is conserved across vertebrates (Supplementary figure 6). This site was located at position 4502-4508 of *FOXP1* 3′UTR cloned on the Gluc-FOXP1-3′UTR-2 construct.

These results showed the presence of a functional miR-92a binding site on the 3′ extremity of the *FOXP1* 3′UTR, and thus demonstrated that *FOXP1* was a target of miR-92a.

### miR-92a, FOXP1, and apoptosis, proliferation and tumorigenicity

**Figure 5:**
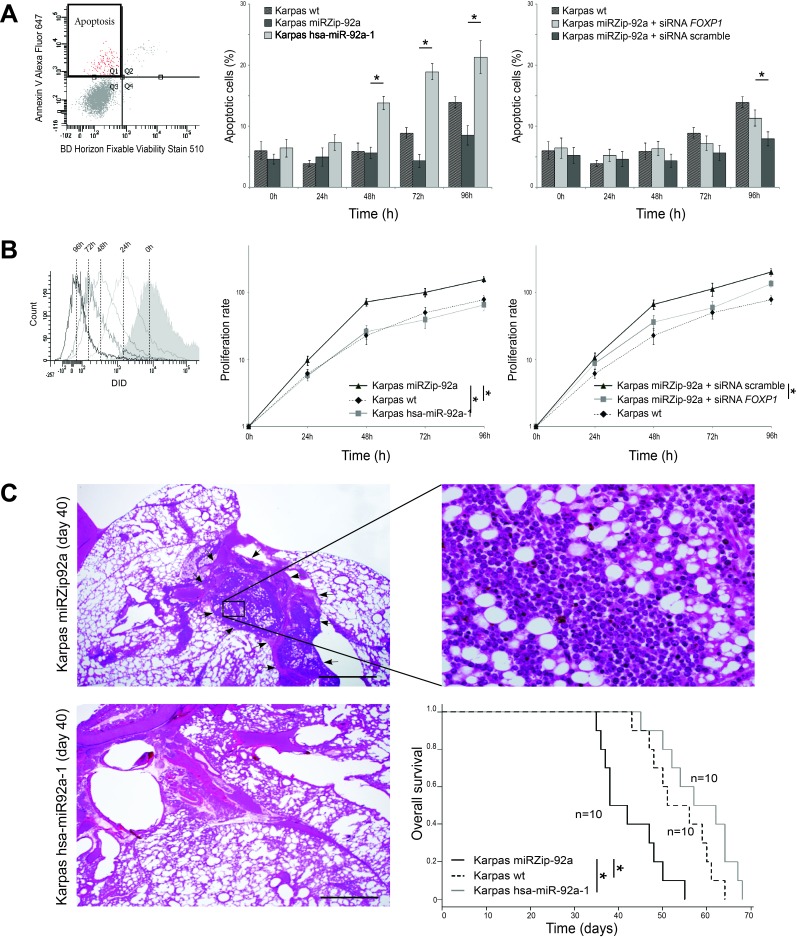
Functional analyses of miR-92a expression in PMBL cell lines The tree Karpas cell lines were cultured for 7 days **A**. Flow cytometry analyses of the two transduced Karpas cell lines show a significantly higher apoptosis rate, using Annexin V test, in Karpas hsa-miR-92a-1 (overexpressing miR-92a) than in Karpas miRZip-92a, at 48h, 72h and 96h. Karpas wt also had a significantly lower apoptosis rate than Karpas hsa-miR-92a-1 cells (under-expressing FOXP1) after 48h, and a higher apoptosis rate than Karpas miRZip-92a cells (overexpressing FOXP1) after 72h (*p* < 0.05). **B**. Flow cytometry analyses of the two transduced Karpas cell lines show a significantly lower proliferative rate in Karpas hsa-miR-92a-1 (overexpressing miR-92a) than in Karpas miRZip-92a, at 24h, 48h, 72h and 96h. Karpas wt has the same proliferation rates as Karpas hsa-miR-92a-1. **C**. Pathological study of the lung and mediastinum performed at day 40 shows an extensive mediastinal infiltration (arrows) of lymphoma cells (higher magnification) in mice injected with Karpas miRZip-92a, while mice injected with Karpas hsa-miR-92a-1 and euthanised at the same date have no mediastinal infiltration. Statistical analyses show a shorter mean overall survival of mice injected with Karpas miRZip-92a cells (40 days) compared with mice injected with Karpas hsa-miR-92a-1 cells (59.5 days)(*p* < 0.05). The mean overall survival of Karpas wt is 53.9 days, significantly longer than the overall survival of mice injected with Karpas miRZip-92a cells (40 days) (*p* < 0.05).

Cytometric analyses of apoptosis showed that, after 48h, Karpas miRZip-92a cells overexpressing FOXP1 had a significantly lower apoptosis rate than Karpas hsa-miR-92a-1 cells under-expressing FOXP1 (Figure 5A). Karpas wt also had a significantly lower apoptosis rate than Karpas hsa-miR-92a-1 cells under-expressing FOXP1 after 48h, and a higher apoptosis rate than Karpas miRZip-92a cells overexpressing FOXP1 after 72h.

When proliferation was studied, Karpas miRZip-92a cells had a significantly higher proliferation rate after 24h than Karpas wt and Karpas hsa-miR-92a-1 cells (Figure 5B). Karpas wt has the same proliferation rates as Karpas hsa-miR-92a-1.

FOXP1 overexpression induced by miR-92a downregulation was rescued using a siRNA targeting *FOXP1* on Karpas miZip-92a cells. Karpas miRZip-92a transfected with siRNA *FOXP1* had a significantly higher apoptosis rate after 96h, and a significantly lower proliferation rate after 48h compared to Karpas miZip-92a transfected with a scramble siRNA.

The tumorigenicity of Karpas wt, Karpas miRZip-92a and Karpas hsa-miR-92a-1 cell lines was assessed after injection of 10×106 cells into three groups of 10 NOD-SCID mice. Statistical analyses showed a shorter mean overall survival of mice injected with Karpas miRZip-92a cells (40 days) compared to mice injected with Karpas hsa-miR-92a-1 cells (59.5 days)(*p* < 0.05). The mean overall survival for Karpas wt was 53.9 days, significantly shorter than the overall survival of mice injected with Karpas miRZip-92a cells (*p* < 0.05).

Comparison of the tumorigenicity of Karpas miRZip-92a and Karpas hsa-miR-92a-1 in tissue was performed on mice euthanised at the same time in two series of 10 mice. These analyses were performed at days 35, 36, 37, 38, 42, 47, 48, 50 and 55. A massive involvement of mediastinal area by lymphoma cells was found in 8/10 mice injected with Karpas miRZip-92a cells but not in mice (0/10) injected with Karpas hsa-miR-92a-1 cells euthanised at the same date (Figure 5C).

## DISCUSSION

We identified here an overexpression of miR-92a in a series of PMBL compared to DLBCL human samples, and, using a combined bioinformatics and transcriptomic approach, we showed that *FOXP1* was the main target of miR-92a in PMBL.

The miR-17∼92 cluster is one of the most potent miRNA oncogenes, located at chromosome 13q31, a region amplified in Burkitt’s lymphoma, DLBCL, follicular and mantle cell lymphoma [31]. Increased miR-17∼92 expression is found in B-cell lymphomas[26] and solid tumors (breast [32], colon [33], lung [23], neuroblastoma [34]). The miRs localized in paralog clusters can also be involved in aggressive lymphoma. They are associated with a high proliferation signature in mantle cell lymphoma [26], and with low survival in cutaneous B-cell lymphoma [35]. The absence of significant difference between PMBL and cHL for the expression level of the miRs in the oncogenic cluster is coherent with the similarities found between the two entities in transcriptional profiles, with high levels of expression of cytokine pathway components, TNF family members and extracellular matrix elements [8]. The fact that the miR from the paralog clusters did not show any significant change when PMBL were compared to cHL or to DLBCL is in favour of an absence of functional redundancy or compensation between the genes studied [21].

Large clusters of miRNAs are often expressed polycistronically. Therefore, it is important to assess the function of individual miRNAs within the cluster [18]. In this study, when we compared expression level of miR-17∼92 in PMBL and DLBCL, we showed a down-regulation of every miRs in PMBL except for miR-92a, that was significantly overexpressed. This had not been reported in the two previous studies of miR expression in PMBL [36, 37]. MiR-92a, overexpressed in our series of PMBL human samples and Karpas cell line, can exert antagonist biological functions in experimental conditions: in the Eμ-myc Burkitt’s lymphoma model, it promotes c-Myc-induced apoptosis [19, 38]; in mouse embryonic fibroblasts, and in primary B-cells, it enhances c-Myc-induced cell proliferation [38].

To study the function of miR-92a in human PMBL, we transduced human Karpas cells to let them overexpress or underexpress miR-92a. We found a significantly higher rate of apoptosis and lower rate of proliferation in cells overexpressing miR-92a. In addition, when these transduced cells were injected into NOD-SCID mice, a significantly higher overall survival, together with a less-severe mediastinal involvement, was observed in mice injected with Karpas cells overexpressing miR-92a.

Overall, these results indicated a tumor-suppressor role of miR-92a in PMBL.

This is coherent with the fact that PMBL, an aggressive lymphoma, has a more favourable outcome than other subgroups of DLBCL: 64% 5 year-survival rate *versus* 46% for DLBCL after anthracycline-combined multiagent chemotherapy [1]; 88.50% 3 year survival rate versus 78.20% for DLBCL after R-CHOP-21 [39].

Using combined *in silico* prediction and transcriptomic analyses, we identified four possible down-regulated targets for miR-92a. 3′UTR assay together with protein expression analysis in transfected cell lines, enabled us to identify FOXP1 as the target of miR-92a in PMBL, a result not previously established.

*In situ* analyses in PMBL and DLBCL human samples confirmed that FOXP1 RNA and protein expressions were significantly lower in PMBL than in DLBCL, in accordance with the higher miR-92a expression found in PMBL than in DLBCL. An elevated *FOXP1* RNA expression level has been reported in DLBCL, activated B-cell like (ABC) subtype [40], and MALT lymphoma at high risk of transforming into aggressive DLBCL [9]. This overexpression of FOXP1 can be caused by a trisomy 3 in ABC-DLBCL [13], and by t(3 ;14)(p14 ;q32) translocation, involving *FOXP1* and *IgH* loci, in ABC-DLBCL and Malt lymphoma [14, 15]. A recent NGS study of 215 B-cell lymphoma characterized 3 mutated genes: *ITPKB, MFHAS1*, and *XPO1* present in 18 cases (39%, 28%, and 39% respectively) of PMBL [7]. A literature analysis did not show any mechanistic link published between these three genes and FOXP1. HL shares a low FOXP1 protein expression with PMBL [13, 41, 42]. In contrast, the high level of FOXP1 protein expression in DLBCL has been included in immunostain algorithms [43–45], which can be associated with FISH tests [46, 47], or with a reciprocal expression of HIP1R protein to classify DLBCL into molecular subtypes with prognostic value in patients treated with rituximab [48].

The role of *FOXP1* in human lymphoma has mostly been studied in DLBCL cell lines. Transcriptomic analyses of ABC-DLBCL *versus* GCB-DLBCL cell lines identified 237 FOXP1 targets [49]. Using this list to filter our transcriptomic datasets of PMBL *versus* DLBCL patient samples, we identified 9 FOXP1 targets: 3 downregulated by FOXP1 (*DERL3, TTC28*, and *TPRA1*), and 6 upregulated by FOXP1 (*FMNL3, DNAJB6, KMO, SNX29, ERP44*, and *CD80*). Since the originality of our study is the use of patient samples that were carefully laser-microdissected to obtain high quality RNAs, we also performed a systematic review of databases presently available on FOXP1 targets in human samples. In the “transcription factor encyclopedia” (http://cisreg.cmmt.ubc.ca/) database, this enabled us to find 8 FOXP1 targets. Two of them were overexpressed in PMBL *versus* DLCBL human samples, as expected. In addition, the upregulation of *CDKN1A*, one of these two FOXP1 target genes is coherent with the clinical data. This gene corresponds to p21 protein whose expression has been reported to be linked to better prognosis in DLBCL [50]. So far, *CDKN1A* upregulation in PMBL has not been reported. It could contribute to the better prognosis of this form of human B-cell lymphoma (Supplementary figure 7).

The ability of FOXP1 to enhance proliferation has been demonstrated using siRNA-mediated silencing [17]. Another recent study showed that FOXP1 directly represses transcription of proapoptotic genes and cooperates with NF-B to promote human B-cell expansion and survival [51]. NF-κB is also activated in PMBL [2, 8, 52], but FOXP1, which we identified as the target of miR-92a, is down-regulated in PMBL, and cannot be involved in the same way as in DLBCL.

The relevance of miR-92a and FOXP1 as new diagnostic markers to better characterize PMBL needs to be tested in a large multicenter study, but the feasibility of their use in daily practice is an important point. Mir-92a can be tested using a simple PCR performed on small RNA fragments extracted from formalin-fixed, paraffin-embedded tissues, and FOXP1 expression can be assessed using common immunohistochemistry on paraffin sections.

In conclusion, we demonstrated the post-transcription regulation by miR-92a through FOXP1 targeting in PMBL.

## MATERIALS AND METHODS

### Patients tissue samples, cell lines

The series included eighty biopsies performed for diagnostic purposes; part of the material remaining after the diagnosis had been established was used for research. The Fundación-Santa-Fe-de-Bogotá-Hospital ethical review board approved the protocol.

Forty patients had newly diagnosed PMBL, and twenty patients newly diagnosed DLBCL not otherwise specified. Twenty other patients had newly diagnosed cHL with mediastinal masses, 10 from Hôpital-Saint-Louis, and 10 from Bogotá-Hospital. Patient characteristics at initial diagnosis are summarized in Table 1.

**Table 1:** Patients characteristics

Characteristics	PMBL n= 40	DLBCL patients n= 20	cHL patients n= 20
Gender M/F	9:31	8:12	9:11
Median age (years) range	27.7 18-47	64.8 26-82	33.4 20-48
Stage I-II III-IV	10 30	10 10	15 5
Extranodal disease	0	11	0
Nb of extranodal sites 0 1 >2	__ __ __	8 7 5	__ __ __
LDH above normal	26	12	6
Performance status 0-1 2	34 6	15 5	17 3
Mediastinal mass Nodal site ≥2 ESR ≥50	40 __ __	0 __ __	20 5 9
IPI score/SPi	0-1 (13) 2 (25) 3 (2) 4-5 (0)	0-1 (4) 2 (8) 3 (2) 4-5 (6)	Early (8) Intermediate (7) Advanced (5) __
Treatment	R-ACEVP (24) R-CHOP (16)	R-CHOP	ABVD

Abbreviations: PMBL, Primary mediastinal large B-cell lymphoma; DLBCL, diffuse B-cell lymphoma; cHL, classic Hodgkin lymphoma; LDH, lactate dehydrogenase; IPI, International prognostic index; SPI, ESR, erythrocyte sedimentation rate, R-ACBVP, rituximab, doxorubicin, cyclophosphamide, bleomycin, vindesine and prednisone; R-CHOP, rituximab, doxorubicin, vincristine, prednisone; ABVD, Adriamycin, bleomycin, vinblastine, dacarbazine

For all eighty patients, formalin-fixed and paraffin-embedded tissue samples were available. Histological diagnosis was performed by 2 pathologists (MR, CS), according to WHO 2008 criteria. Snap-frozen tissue samples were also available for 8 PMBL, and 9 DLBCL. Laser-microdissection of tumor cells was performed on all samples studied, whether formalin fixed or snap frozen, by two trained pathologists (MR, AJ).

This study also included two human cell lines: a PMBL cell line (Karpas-1106P, Sigma, France) and a DLBCL cell line (SU-DHL-5, DSMZ, Germany). Cell lines were maintained under standard culture conditions: 37°C and 5% CO2 in RPMI-1640 medium supplemented with glutamine (200mM), 20% fetal-calf-serum (GE-Life-Sciences/PAA-Laboratories, Inc), and 0.5mg/mL penicillin-streptomycin (Invitrogen, France).

### Lentiviral infection of cell lines

Karpas wt cells (25×10^4^ per milliliter) were transduced with VSV-G pseudotype viral particles (Ozyme, France) (MOI_(Karpas)_=90) that included miRZip-92a anti miR-92a construct (Ozyme, France) on RetroNectin precoated dishes (Takara, Ozyme, France) to induce a low expression of miR-92a. A high expression of miR-92a was induced by the inclusion of hsa-miR-92a-1 pre-miRNA construct (Ozyme, France) on RetroNectin precoated dishes.

After 24 hours, the stably transduced clones were selected by puromycin (0,1 ng/ul).

Transduction efficiency was assessed using green fluorescence protein on a FACSCalibur cytometer from Becton-Dickinson. miR-92a expression levels were measured using RT-qPCR (Supplementary figure 1)

### Quantification of expression of the miR-17∼92 cluster and its paralogs

Laser-microdissection was performed on each tissue sample using a PALM-Microbeam/Zeiss system (Zeiss, Wetzlar, Germany). On 7-μm-thick sections of formalin-fixed paraffin-embedded samples, tumor cells were identified using anti-CD20-antibody (Clone L26, Dako, France) for PMBL and DLBCL, and anti-CD30-antibody (Clone Ber-H2, Dako, France) for cHL. In each PMBL and DLBCL sample, approximately 1500 tumor cells were microdissected, corresponding to a mean area of 301.440 μm2 (PALM-Robot-Software). In cHL approximately 1000 tumor cells were microdissected, corresponding to a mean area of 490.625 μm^2^.

Total RNA was extracted using RecoverAll-isolation-kit (Ambion, Cambridgeshire, UK), according to the manufacturer’s protocol. RNA quality was assessed by spectrometric assay (NanoDrop^®^, Thermo-scientific, USA).

For human cell lines, 10×10^6^ cells were collected for Karpas wt, Karpas miRZip-92a, Karpas hsa-miR-92a-1 and SU-DHL-5 wt. Total RNA was extracted using mirVana miRNA Isolation Kit (Ambion, Invitrogen, France), according to the manufacturer’s protocol. RNA quality was assessed by spectrometric assay.

The miR-17∼92 cluster and its paralogs (miR-106a-363 cluster and miR-106b-25 cluster) were analyzed in all 80 formalin-fixed paraffin-embedded samples and two cell lines by Q-RT-PCR using miRNA-reverse-transcription-kit, and hsa-miR-17, hsa-miR-18a, hsa-miR-18b, hsa-miR-19a, hsa-miR-19b, hsa-miR-20a, hsa-miR-20b, hsa-miR-25, hsa-miR-92a, hsa-miR-93, hsa-miR-106a, hsa-miR-106b, hsa-miR-363 Taqman assays (Applied-Biosystem, Darmstadt, Germany) on Biorad Real-Time Detection System (Bio-Rad, CA, USA). RNU24 and RNU44 assays were used as endogenous controls for normalization. Data were then normalized to the mean of the two references genes, using CFX manager software (Bio-Rad, France), and expression levels were calculated using 2^-ΔCq^ method.

### Microarray analyses and miR-92a target prediction

Laser-microdissection was performed in cryopreserved samples available for 8 patients with PMBL and for 9 patients with DLBCL. Seven μm-thick cryocut sections were incubated for 5 min with anti-human-CD20 antibody (clone L26, Dako, France) labeled with APEX™ Alexa Fluor^®^ 488. Immunofluorescent tumor cells were microdissected using PALM-Microbeam/Zeiss system. In each case, quantification was performed using PALM-Robot-software: approximately 1500 tumor cells for a mean 301.440 μm2 area.

For the wild type and the two transfected Karpas human cell lines studied, 10×10^6^ cells were used.

Total RNA extraction was performed in human samples and cell lines as described above. RNA quality was checked using Agilent Bioanalyzer (Agilent-Technologies, CA, USA). All samples had RNA integrity number (RIN) between 7 and 8,3. Fifty ng of total RNA were amplified and labelled using the Low-Input-Quick-Amp-Labeling-kit and hybridized overnight in Agilent-Whole-Human-genome-Oligo-Microarrays-8×60k. Scanning was carried out with Microarray-Scanner-System. Intensity profile for each probe set was calculated using Agilent-Feature-Extraction-Software.

For gene analyses, comparisons were performed for between PMBL and DLBCL patients and between the four human cell lines studied.

To identify genes with significant expression changes, analyses were performed in the R-statistical-computing-environment (version x64 3.0.2, http://www.r-project.org). The Bioconductor Agi4×44Preprocess package was used to read Gene-expression-assay data files, to apply a background correction, to filter probes and to normalize gene expression across samples. The SAMR package was used to identify differentially expressed genes in samples with the two-class unpaired method, based on the ExpressionSet generated by the Agi4×44-Preprocess package. Hierarchical clustering was performed with the heatmap.2 function of R, evaluating distances between samples and genes using the Manhattan method and reordering them with Ward’s algorithm.

We then determined whether there was an association between differentially expressed genes in samples and biological pathways using GAGE package. We also looked for biological pathways with a significantly different expression in PMBL *versus* DLBCL, and in the four human cell lines studied.

The microarray data set is filed in the ArrayExpress-public-database at the European-Bioinformatic-Institute (E-MTAB-4165 / GAPIHAN / primary mediastinal large B-cell lymphoma).

*In silico* target prediction for miR-92a was carried out using the CoMeTa tool (Co-expression Meta-analysis of miRNA Targets, http://cometa.tigem.it). We obtained a list of putative miR-92a targets. We then studied whether they were differentially expressed in PMBL *versus* DLBCL. False discovery rates (FDR) associated with p-values were calculated using the p.adjust R command (Benjamini and Hochberg method). Associations with FDR-adjusted p-values (q-values) of less than 0.05 and fold changes of more than 2 were considered significant. The same comparisons were carried out for human cell lines with low expression of miR-92a (Karpas miRZip-92a and SU-DHL-5 wt) *versus* human cell lines with high expression of miR-92a (Karpas hsa-miR-92a-1 and Karpas wt).

### Analyses of *FOXP1* expression

Quantification of mRNA expression of putative miR-92a targets was performed from available snap-frozen samples from 8 patients with PMBL and 9 with DLBCL, and from the wild type and the two transfected Karpas cell lines, with Q-RT-PCR using GoScript™-Reverse-Transcription System (Promega, France), GoTaq^®^ qPCR Master Mix (Promega, France), and taqman primers and probes for *FOXP1* (Hs00544877_m1), CIC (Hs00943425_g1), *SMG1* (Hs00247891_m1), and *INADL* (Hs00195106_m1). Assays were read on the Biorad Real-Time Detection System. *TBP* (Hs99999910_m1) (Life-Technologies) was used as the endogenous control for normalization. Data were normalized on the reference gene, using CFX manager software and expression levels were calculated using the 2^-ΔCq^ method.

Immunohistochemistry was performed on 5μm-thick snap-frozen sections from patient samples, and smears from the four cell lines studied, using anti-human FOXP1 antibody (HPA003876-100UL, 1/10, Sigma, FRANCE). Controls systematically included absence of primary antibody and incubation with an irrelevant rabbit polyclonal antibody. Tissue sections were analyzed under Olympus-AX-70 microscope with a 0.344mm^2^ field size at X400 magnification (Olympus, Tokyo, Japan). Counts of positive FOXP1 cells were performed on five different fields by two independent observers (MR, AJ). Results were expressed as means +/- standard deviation.

### *FOXP1* copy number analysis by droplet digital PCR

Laser-microdissection was performed for the 8 PMBL and 9 DLBCL samples that had already been studied for transcriptomic analyses. On 7 μm-thick cryocut sections, tumor cells were identified using anti-CD20-antibody. In each case, approximately 1500 tumor cells were microdissected for a mean area of 301.440 μm^2^.

For the SU-DHL-5 wt, Karpas wt, Karpas hsa-miR-92a-1 and Karpas miRZip-92a human cell lines studied, 10×10^6^ cells were used.

DNA was extracted using QIAamp-DNA-Mini-kit (Quiagen, France) and DNA quality was assessed by spectrometric assay.

Droplets were generated in a QX100 droplet generator (Bio-Rad, CA, USA). PCR was performed on a C1000 PCR thermal cycler (Bio-Rad, CA, USA) using FAM-labeled *FOXP1* taqman primers (Hs04751460_cn) and a VIC-labeled copy number reference RNaseP (Life-Technologies, France). PCR results were read in a QX100 droplet reader (Rio-Rad, CA, USA).

### Western blots

10^7^ cells of each of the four cell lines studied were lysed in 200μL lysis buffer (0.5M Tris-HCl, pH 6.8, 2mM EDTA, 10% glycerol, 2% SDS and 5% b-mercaptoethanol). Whole lysates (20μg) were separated on a 10% SDS-PAGE and immunoblotted using anti-human FOXP1 (HPA003876-100UL, 1/2500, Sigma, FRANCE) or anti-human beta-actin (AC-74 clone, 1/5000, Sigma, France). Bands were visualized by chemiluminescence on an LAS-3000 analyser (Fujifilm, France) and analyzed using Multi-Gauge-V3.0 software (Fujifilm, France).

### Luciferase reporter assay

*FOXP1* 3′UTR sequence was inserted downstream of the secreted Gaussia luciferase (GLuc) reporter gene. The GLuc vector system had been driven by SV40 promoter for expression in mammalian cells.

To cover the whole *FOXP1* 3′UTR, two plasmidic constructions: Gluc-FOXP1-3′UTR-1 and Gluc-FOXP1-3′UTR-2 (GenoCopoeia, Tebu-bio, France) were necessary. Each of them had an identical 72bp sequence to form an overlap.

To perform this Luciferase reporter assay, we chose the 293T-cell line because it had already been efficiently transfected with miR-92a [30].

293T-cells were first transfected with these constructions using lipofection (Ozyme, France). The same cells were in a second step transfected with 50nM of hsa-miR-92a-3p mimic (Exiqon, France) using lipofection. Non-transfected 293T-cells were used as controls for plasmid transfection. The negative control for miR activity was cel-miR-39-3p.

Luciferase activity was measured 72 hours after miRNA transfection using the Secrete-Pair-Dual-Luminescence-Assay-Kit (Genocopoeia, Tebu-bio, France). Analyses were performed on a FLUOstar Optima spectrophotometer (BMG Labtech), using the Secreted alkaline phosphatase (SEAP) signal to normalize the GLuc activity (GenoCopoeia, Tebu-bio, France).

### *FOXP1* siRNA downregulation

5×10^6^ Karpas miRZip-92a cells, cultured in a 6-well Petri dish, were transfected with *FOXP1* siRNA (sc-4483, Santa Cruz Biotechnology, France) or scrumble (siRNA sc-37007, Santa Cruz Biotechnology, France) in siRNA transfection reagent (sc-39528, Santa Cruz Biotechnology, France) according to the manufacturer’s protocol.

### Proliferation of transfected cells

5×10^6^ cells of each wild type and the two transfected Karpas cell lines studied were uniformly labeled in Vybrant^®^ DiD cell-labeling solution (Invitrogen, France) and cultured in a 6-well Petri dish for 7 days. Cells were analyzed on a BD-Canto-II-cytometer each day at wavelength 660/20nm mean fluorescence. Proliferation rates were calculated according to the fluorescence decrease.

5×10^6^ cells of each wild type Karpas, Karpas hsa-miR-92a-1, Karpas miRZIP-92a, Karpas miRZIP-92a + siRNA *FOXP1* and Karpas miRZip-92a + siRNA scramble cell lines were uniformly labeled in Vybrant^®^ DiD cell-labeling solution (Invitrogen, France) and cultured in a 6wells-Petri-dish for 7 days. Cells were analyzed on a BD-Canto-II-cytometer each day at wavelength 660/20nm mean fluorescence. Proliferation rates were calculated according to the fluorescence decrease.

### Apoptosis of transfected cells

5×10^6^ cells of each wild type and the two transfected Karpas cell lines were cultured on a 6-well Petri dish for 7 days. Each day, 1×10^6^ cells were labeled using 5μL Annexin V-647 (Invitrogen, France) and 1μL BD-Horizon-Fixable-viability-stain-510 (BD, France). Analyses were performed using flow cytometry at wavelengths 510/50nm and 660/20nm respectively.

5×10^6^ cells of each wild type Karpas, Karpas hsa-miR-92a-1, Karpas miRZIP-92a, Karpas miRZIP-92a + siRNA *FOXP1* and Karpas miRZip-92a + siRNA scramble cell lines were cultured on a 6wells-Petri-dish for 7 days. Each day, 1×10^6^ cells were labeled using 5μL Annexin V-647 (Invitrogen, France) and 1μL BD-Horizon-Fixable-viability-stain-510 (BD, France). Analyses were performed using flow cytometry at wavelengths 510/50nm and 660/20nm respectively.

### *In vivo* tumorigenicity of transfected cells

All experimentations were performed in accordance with the European Community recommendations (2010/63/UE).

Three groups of 10 six-week-old NOD-SCID female mice (Janvier lab, France), maintained in specific pathogen-free conditions were used. Each group had intravenous injections of 10×10^6^ Karpas wt, Karpas hsa-miR92a-1 or Karpas miRZip-92a cells. A clinical score was assessed daily on weight loss, grooming, posture, respiratory rate and activity for each mouse. Animals were euthanised using cervical dislocation when protocol limit-breaks were reached, and systematic pathological analyses were performed.

To compare tumor extension in the different organs for Karpas miRZip-92a and Karpas hsa-miR-92a-1 cell lines at the same time-point, two other groups of 10 six-week-old NOD-SCID female mice had similar injections. Since Karpas miRZip-92a mice were the first to die, whenever one mouse in this group died, a mouse from the Karpas hsa-miR-92a-1 group was also euthanised and systematic concomitant pathological analyses were performed.

### Statistical analyses

Analyses were performed with GraphPad and SAS 8.2 software (SAS Institute Inc, USA).

MicroRNA expression levels were expressed as the median fold change +/-Standard Deviation. mRNA expression levels were expressed as the mean fold change +/-Standard Deviation.

The Mann-Whitney test was used to compare expression levels in PMBL, DLBCL and cHL samples, and in the four cell lines studied.

The results of these comparisons were considered significant when the two-sided *p*-value was <0.05. This significance threshold was corrected for multiple comparisons using the Bonferroni method.

Droplet-digital-PCR data were analysed using the QuantaSoft software to measure the fraction of positive droplets and calculate the amount of template per droplet based on a Poisson distribution.

## SUPPLEMENTARY MATERIALS FIGURES


