# Supplementary material for: Primary mediastinal large B-cell lymphoma: transcriptional regulation by miR-92a through FOXP1 targeting
Source: Oncotarget. 2016 Oct 28;8(10):16243–58. doi: 10.18632/oncotarget.12988 (PMC5369960; doi:10.18632/oncotarget.12988)
Supplement: Supplementary file 1 [file oncotarget-08-16243-s001.pdf]

Primary mediastinal large B-cell lymphoma: transcriptional regulation by miR-92a through FOXP1 targeting

Supplementary Material

Supplementary figure 1

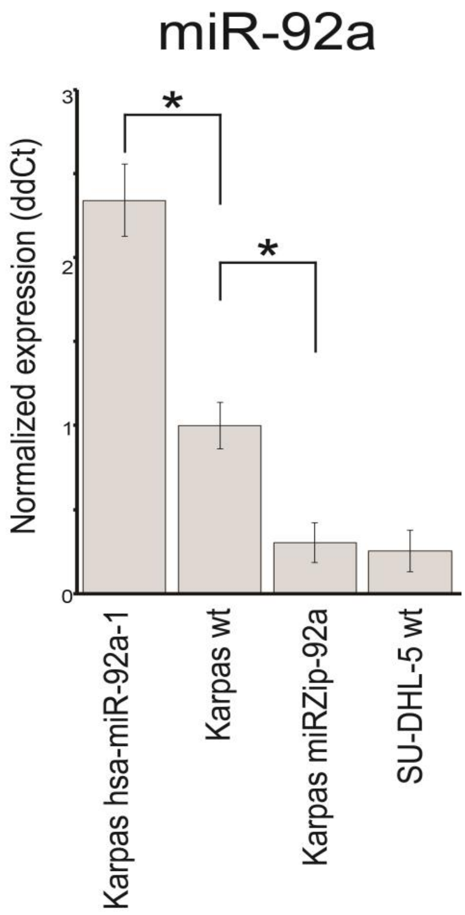

Supplementary figure 1

miR-92a expression level assessed using Q-RT-PCR in Karpas wt, Karpas transduced cell lines and SU-DHL-5 wt shows that Karpas has-miR-92a-1 overexpress miR-92a and Karpas miRZip-92a underexpress miR-92a. Results are expressed as mean fold change +/- standard deviation. Statistical analyses were performed using the Mann-Whitney test. *P=<0.05.

Supplementary figure 2

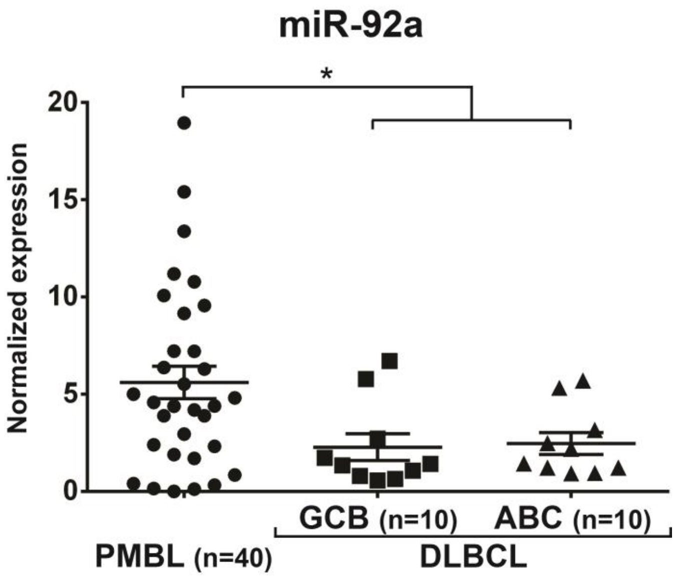

Supplementary figure 2

MiR-92a expression level assessed using Q-RT-PCR in 40 PMBL and 20 DLBCL shows a significantly higher expression level in PMBL than in DLBCL. In this series, there is no significant difference in miR-92a expression level between GCB and ABC DLBCL subtypes. *P=<0.001.

Supplementary figure 3

miR-17-92 (13q31.3) (Oncogenic cluster)

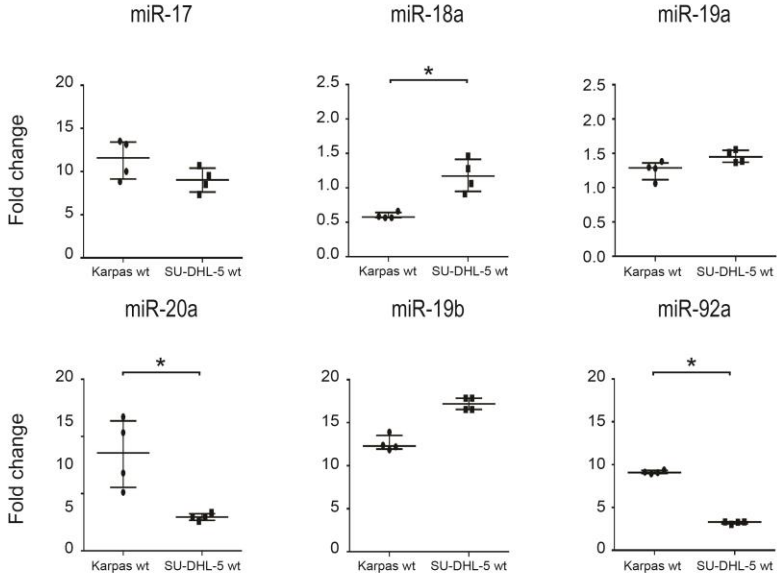

miR-106a-363 (xq26.2) (Paralog)

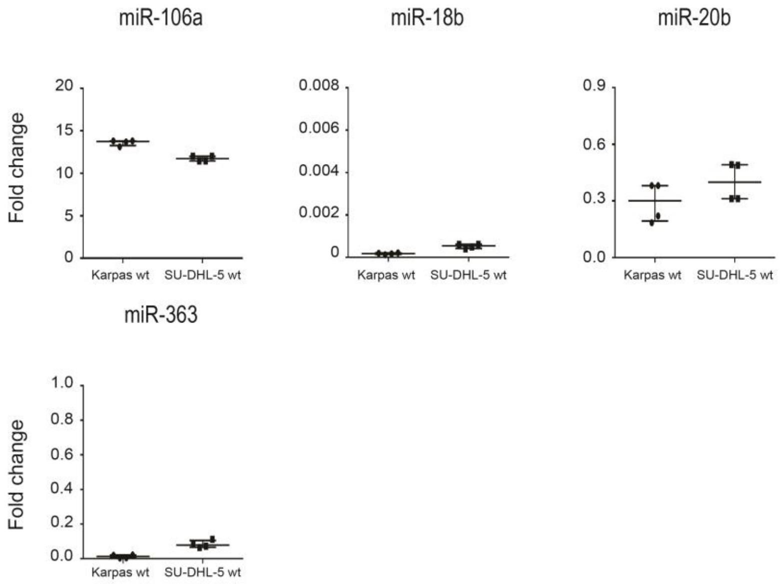

miR-106b-25 (7q22.1) (Paralog)

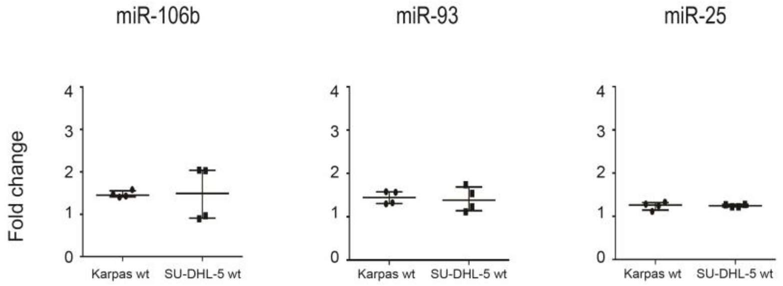

Supplementary figure 3

Quantification of expression levels for each microRNA of miR-17~92 cluster and its paralogs in Karpas wt and SU-DHL-5 wt human cell line shows a significantly higher expression level of miR-92a in Karpas wt than in SH_DHL-5 wt.*P=<0.001.

Supplementary figure 4

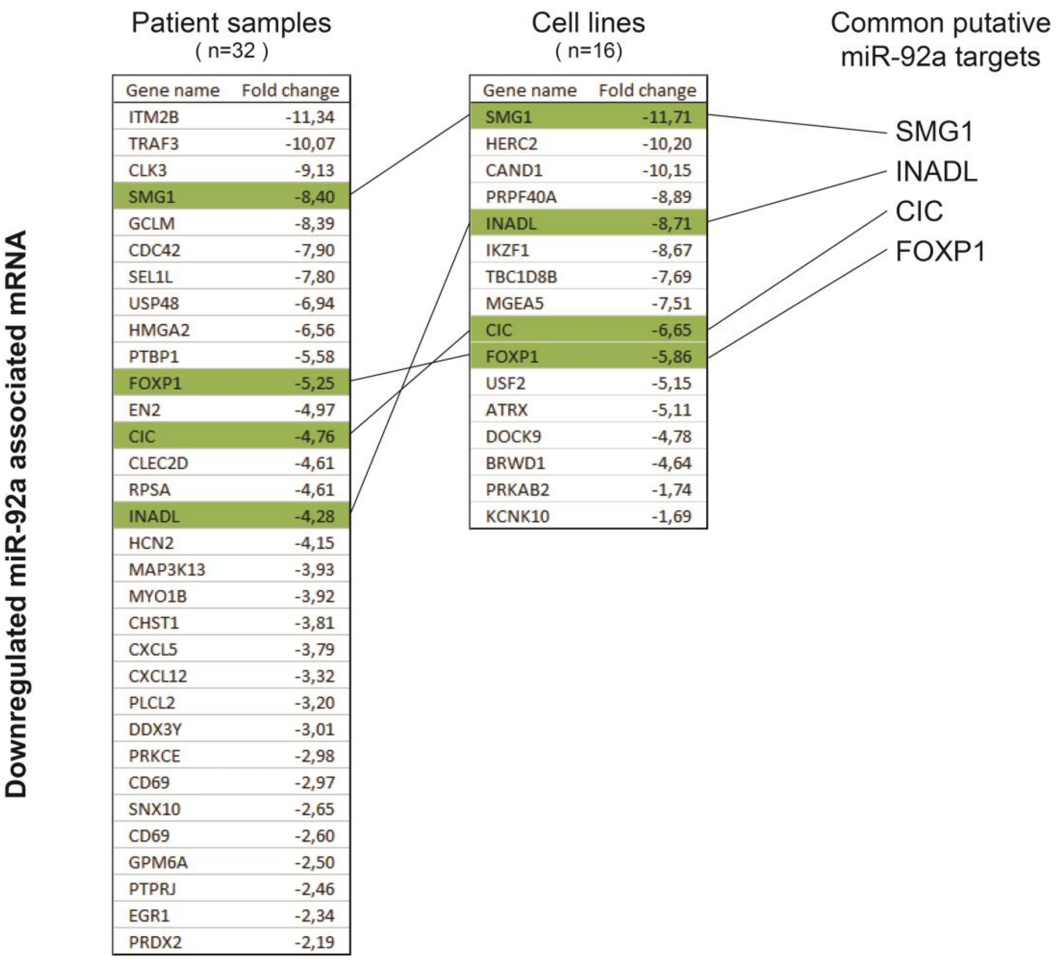

Supplementary figure 4

Filtered gene-lists comparison of PMBL versus DLBCL identifies 32 down-regulated miR-92a targets (FDR<0.05, fold-change>2, q-value<0.05).

Comparison of miR-92a up-regulating versus miR-92a down-regulating cell lines identifies 16 down-regulated miR-92a targets (FDR<0.05, fold change>2).

Patient samples and cell lines have 4 common down-regulated miR-92a targets: *CIC*, *FOXP1*, *INADL*, and *SMG1*.

Supplementary figure 5

Upegulated miR-92a associated mRNA

Patient samples
(n=17)

Gene name	Fold change
DYNLT3	6,51
DYNLT3	5,19
C21orf91	5,00
PDLIM7	4,91
CAMKK2	4,78
ZNF365	4,25
DOCK9	4,12
LMO2	4,06
TEAD1	4,02
DOCK9	3,94
CALM3	3,79
TBX3	3,67
CLIC4	3,32
SIPA1L1	3,28
CHST7	3,16
EPB41L4B	3,11
EBP	3,10
DPT	3,03
NEDD4L	2,83
MGLL	2,76
TRIO	2,70
DACT1	2,58

Cell lines
(n=3)

Gene name	Fold change
PAX3	7,80
ATF3	2,16
PFKFB4	2,12

No upregulated
miR-92a associated mRNA

Supplementary figure 5

Filtered gene-lists comparison of PMBL versus DLBCL identifies 17 up-regulated miR-92a targets (FDR<0.05, fold-change>2, q-value<0.05).

Comparison of miR-92a up-regulating versus miR-92a down-regulating cell lines identifies 3 up-regulated miR-92a targets (FDR<0.05, fold change>2).

Patient samples and cell lines have no common up-regulated miR-92a target.

Supplementary figure 6

Human FOXP1 ENST00000318789.4 3' UTR length: 6428

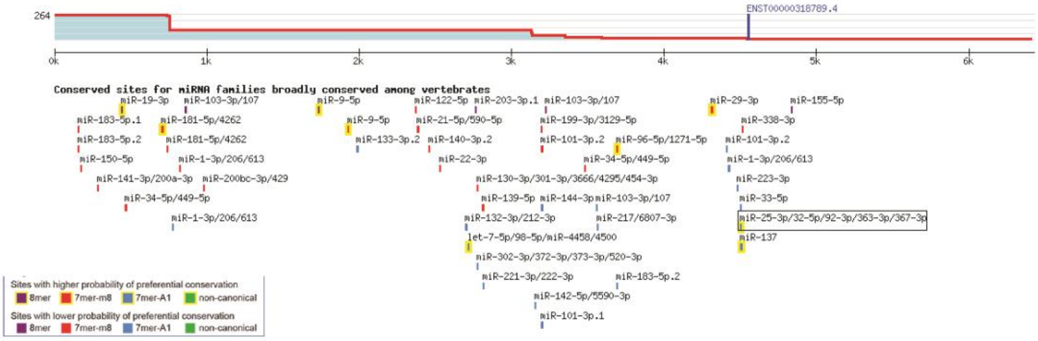

Conserved miRNA binding sites across vertebrates

	Predicted consequential pairing of target region (top) and miRNA (bottom)	Site type	Context++ score	Context++ score percentile	Weighted context++ score	Conserved branch length	P_{CT}
Position 4502-4508 of FOXP1 3' UTR<br>hsa-miR-367-3p	5' ...AUAAGCAUUCUCAAA-UGCAAUAA...<br>3' AGUGGUAACGAUUUCACGUUAA	7mer-A1	-0.17	89	-0.02	4.693	0.77
Position 4502-4508 of FOXP1 3' UTR<br>hsa-miR-363-3p	5' ...AUAAGCAUUCUCAAAUGCAAUAA...<br>3' AUGUCUACCUAUGGCACGUUAA	7mer-A1	-0.14	86	-0.02	4.693	0.77
Position 4502-4508 of FOXP1 3' UTR<br>hsa-miR-25-3p	5' ...AUAAGCAUUCUCAAAUGCAAUAA...<br>3' AGUCUGGCUCUGUUCACGUUAC	7mer-A1	-0.13	86	-0.01	4.693	0.77
Position 4502-4508 of FOXP1 3' UTR<br>hsa-miR-92a-3p	5' ...AUAAGCAUUCUCAAAUGCAAUAA...<br>3' UGUCCGGCCCUGUUCACGUUAU	7mer-A1	-0.11	85	-0.01	4.693	0.77
Position 4502-4508 of FOXP1 3' UTR<br>hsa-miR-32-5p	5' ...AUAAGCAUUCUCAAAUGCAAUAA...<br>3' ACGUUGAAUCAUUACACGUUAU	7mer-A1	-0.13	85	-0.01	4.693	0.77
Position 4502-4508 of FOXP1 3' UTR<br>hsa-miR-92b-3p	5' ...AUAAGCAUUCUCAAAUGCAAUAA...<br>3' CCUCCGGCCCUGCUCACGUUAU	7mer-A1	-0.10	84	-0.01	4.693	0.77

FOXP1 3'UTR plasmidic constructions

Gluc-FOXP1-3'UTR-1

```
>HmiT067349 (fragment a)
CTATCGGGGCGGGCCAACCCCGAGAATGAAGATTGGAAAAAGGAAAAAAAAAAAAAACACGTCAAAAGTTAGCAGTGAAATTGTTCTCCATTTGTTGTACA 100
GTCTGGAGGATTTTCACTACGTTTTGACAACTCTGAAATGTGTTAACTCTTAGTGCCATCAAGAACCCCATTTGGGAGTATTTTTGATTTTTCTACTTTT 200
TGTTGAAAAAAGGAATTTGTACTCTGTGCATTGGATGGACTTGTTTGGTACTTGGGATTTTCCTCTCTTAACCGTCAACATCAGTGTTGTAAATTTGCTA 300
AACTGATTCACTTTTAGCAGCAGACTTTGAACTGCAGTCCTGCCAACGTTGGACACTGAGGACGCCCGACAGAGCTTGTGCACCTAAGCTGCAGACCAAG 400
CCTTTGCCCAGAATTTAAGGATTCCAATGGACGACCTATTTGCACAGTACTGCATGTTGATTATCACTGCCTTTACTCCTTTTTTTTTTTTTTTTTTTTT 500
TTTTTTTTTGCTTCCAGTTGGGATGGGGAAGGCCTTTGTGTGTGTATTGGGGGGAGGGGTTAAAAAATAATTATCCCAAACTTTTTAATGTATTGCTTTT 600
TTTTTTTTTTTTTTTTCCTTCTACTATACCATTTAAGTTCTGACCTCAGGCCTCCATTTGGGCCGATGGCCTCTTGGAGGCTTAAAGTTTTCTGTACCTT 700
GTGATGAATGTTAATAGGTGTTTTTATTATACAAAGCTGAATGTCATTTCTCGTTTGTAGCTTTCTGTCACTCATTCCATCTTCCTTCAGACATCACCAC 800
GTTTCTCTAAAGTCAGAAAACATTCCGTTTTGGTCTTTTTCAAAAAGGTCCCAAATGCTGCACTCTACACATGAAGGCCCTCTCACACAGACGTGACGTC 900
CTGCCAGAAAGAGAATGAATGACAGAAAAAAAAAAAGAGAGACAAACTCTAGGAACAATGCCGATTCATTCCACGCAGCAGTATTGGGGGTGGTTCGGGGG 1000
AGGGGTGTTTCGGATTTTCTTTTTTTCTTTTCTTTTCTTTTTTTTTTTTTGCAGCAACCATTAATAAATGCCACCACATTCTACCAGCACAAGGAAACAT 1100
AGGCAGCACTGAAAAAAAAAAAAAAAGCTCATATTAATTAGACTGACAATATGGCCTTGGAAGGCTCTCCCTTGTGGAACCAAGTTGCCATGGGCCTTGGG 1200
TGCTCTGCGATAACGGGTGTGGGTTGGTTTTGTTTGCAAAATGGCCAAAAAAAAAAAACCGGCTTCCCCGAGCAGCTGCCCTGAAAGTAGGGGTGGCGGCG 1300
GCGGCGCTGAGTTTATACATTAGTTCAGACCTACTTGGTGGCATTAAACTGTTTGAATGCAAATTCGATTTCAGATTGAACTTGTTAAGGGAGTTAACGA 1400
GGGCTGAGTTCAGCAAATGCTAAAGTGTTAATTTCAAATATGCAAATTTGGTACTGCAGTTTGTTATGCAATATTATATCACCAACCCAGTATCACAAAA 1500
ACTCATAGAAGATATCATGTAGGCCCTGGGCTTTGGGGGGGTCCCAAACATGGTATGCAGAAATGTGATGGTTACAGGTCAGTACAACCTCAGTCCTTAG 1600
AACCCCTCCACACTTCAGCTCTGCACCCACTTTCCTGTCATTTATTTATATAGGACTGTAGTTTTTTTAGTTCGAGAGCCTTTCGAAGCTTAATTTATA 1700
TTCTTTCTTTGTACCTTTTTTCTAAAATTACCAAAGATATTACACAAAGGTAAATTATGTTCTCTGTTTTATGCTTTATCTGATGAAGCCAAATATCCTC 1800
TTATTGTTGATCAAAGGAGGCAAAAGAATTTAGAGGCAAATGACAAGCGATAGGCTATTGCAACCTGAGAAAGAGAACTGCTCCTTCATCGTAAATTTAG 1900
AAGACCAAGTAGATAATGGAACCAAAGTTGTTACTTTTTTCTAGTAGTTATTTTTCCTTTTTCTTTTTGTGTACCTCTACAGAGACCAAAACTCATTCTC 2000
TTAAAGAGATTTTATGGGGCTACTGCAGATAAAAATAGGACACAATATTAAAGGAGCTACAGAAGGAAGGGAGTCCCATCTCATGCAATAAAAAAAAAAT 2100
GAATGTAGCCACTGCAATTAGAGTATCCAATAAAGGAGACAGTTTAGAGTCAGGACAGAAAAGCTTCCATAATTGAACTAGATTACATAATAGTATTTCT 2200
AGAAAAAGAGATATTTTTAGATTGTATGCCACTTTTGTTTAAGAACTGTGCTGTGATCACTGTATTAATTTTGGTTTATCTTGGCATATATCCTTCAGTT 2300
TGTTTTTATTTTTAATTTTTCCTTTTTTTCCGATTAGGCTTTGGTCAGCATTTTTCATTTAAAGAAAAGTAACACTCCCATCCACTCATAAGCTT
```

Gluc-FOXP1-3'UTR-2

```
>HmiT067349 (fragment b)
TTTTTTCCGATTAGGCTTTGGTCAGCATTTTTCATTTAAAGAAAAGTAACACTCCCATCCACTCATAAGCTTGGTACAAAAACTTCTCTGGCAGTTACTT 100
TTGAAGCTTCACTCTGCTTTCTGTATAAAGGGCAGTCTGTGGTCACGCAAGACTTTAAAAAAAAAAAAAAAAAAAAAAAAAAAAAAAAAAAAAAAAAAACTT 200
TTCCAGGCAGCTTCATGATGTGCAGGCAGTAGCCAGACAGGGTCATGGGAAGGGGGCCCTGTGCTTCTAAACTGAGTGGTTGCTGGTTAGTTTGGTATTC 300
AAAAGAGGATAAAAATCTGGTAGATTAGTTCATTCTCAGCATGTGTAGCTAGACATGAGTAAAGATAACAGCATGAGAAACTGTTAGTACGCATACCTCA 400
GTTCAAACCTTTAGGGAATGATTAAAATTTAAAAAAAAAACATTTCACTCAGTTGCACTTAGTCGTATGTCTTGCATGCTTAGTCTAAAGACTGTAGCAA 500
AAAAAAAAAAAAAAGAAAAATTAGATTTACATATCTTTGCAGGTATCACAGCCTTGCAGAAGAACCAACTGAAAAAAAAATTCTCAGGCTTTACAGCAA 600
GCAAACTTCACTATGATTTTTACAATTCTGATTCTGTATCCCCTGGGGGTTATCCCAGTTGCTTCTTTAGGATGGGGTTTATTACGTTGTACATATATCC 700
CGATGTGTCTGTGTGAATCTTTGTCTTTTTTGGGGGAGGGCAGAGGGCGGTTCTTTTTTTAGATATTGTTCCTAAAAAGGAATAAATGCATACACCTGTT 800
TGTCAAAACACCTTTGCTTTTTGTGCAACTGCTTTATATTAACGATACTAAAAAAAAAATAGCTTTGGAAAAAAAAACTACTGTATGTAACGGAATTGCAGA 900
ATATGCTGCACATGTATTTTATTTAGTTATCCTTGCTTTAAGAATATTGGATGACATTTCCTGACATGTGGGAGGGAGAAACTCCCTAACTTTTTTTTTC 1000
TGCTTTTAAACTGTAACATAGTTGAAGATTTCTTTTTTCTGTTCTCATTGATTGGAGCATTTTGTACAGGTTTTGTGTGTGTGTGTGTGTGTGTGTGCGC 1100
GCGTGCGTGTGTGTTAATCTGTTTTTTGATACATTCCTATCCCTTGTGTTTATCCTACCACTGCCTTCCTGGCTATCTTAAACAAGTTCATACATTTGAA 1200
AAGAAAAAAAAATGTTGTTTAAAAAATGTTTTCTCCTGCTGCAGTAAATATTTTGCATGATGAAATTCCAGGGTCACACTTTTCCAAGTTTATCAGTGAA 1300
GTAGTGATTAACAATGGGGAGTGTCAAAACTATTGAACTTTTGTATAAAAAAAAAAAAAACTTTACAAGGTGCCAAGATGTAAAGAAAATCTGTTACTTTT 1400
TTTTTCTCAAAGAAAAGCATACATTAGGGAGGTAGTCCCGTGTGTCAGACAAATGCACTGTCAGGAATGAGGATCCAACCTACCTGTCCCTAGAGTCCGT 1500
CTTGTAAGATGAGTTAGGCTGCCCCTTGGACCAGCCACAAAATGGAATATCAAGGCTTATGTACATACGTGAAGAGTTACCACCAGTCCTGCCACCTTTG 1600
GACAGCTCTAACACCATCCCCAGCATCCAGTCAGACCTAGTAAAGAAAACCTTGGATTCTTAACCCAAGATAGGCTGTAAATCACTAGCTTTTTTTTCCT 1700
CATGAAAAAAAATAGAGTTAAAAAATATTTCCTCTCTTTTCCATATTCCAGCTGAACTCCGTTTCCAAAGGCACAAAGAAGAGTGTGCTTATTCAGATTT 1800
TGAATCTTTTTGGTACCTTTTGGTTAATGACATAGCCTCCTGAAATTCTGGATGTCTTCAAAGTCAGTTTTGCTTCTTTATCCTGAAAATCAGATTTACA 1900
ATGCTGAAGGCATTTCTTGGGCCCAGTGTAGCTCACGCAATCTCTGCTACCCATAAGCCTTGATGAAGATGATACAGTCCGGACTGTGAGCATGGTGCTT 2000
CATGTATATGTGCTGCCAGTAACAAGAATTTTTTTGTTTTGTTTTGTTTTGTTTTGATAAGGCATAAAAGAAACTCATTCCTTGACATCAACTGTAATTC 2100
CATCATTCCATGTCTGCGGATACAGACAATAAAAAAAAATGTTGTGTAGTCAGTACTAATTACTGACATTATAAGCATTCTCAAATGCAATAAAAATGCTG 2200
GTTGTTCACGCTGGT
```

Supplementary figure 6

We used the miRNA target prediction database TargetScan to identify a 7mer-A1 miR-92a-3p binding site on the *FOXP1* 3'UTR, which is conserved across vertebrates. This site is located at the position 4502-4508 of FOXP1 3'UTR. To cover the whole *FOXP1* 3'UTR, two plasmidic constructions: Gluc-FOXP1-3'UTR-1 and Gluc-FOXP1-3'UTR-2 were necessary, each of them with an identical 72bp sequence (in red) to form an overlap. The miR-92a-3p conserved binding site is located in the Gluc-FOXP1-3'UTR-2 sequence (marked in green).

Supplementary figure 7

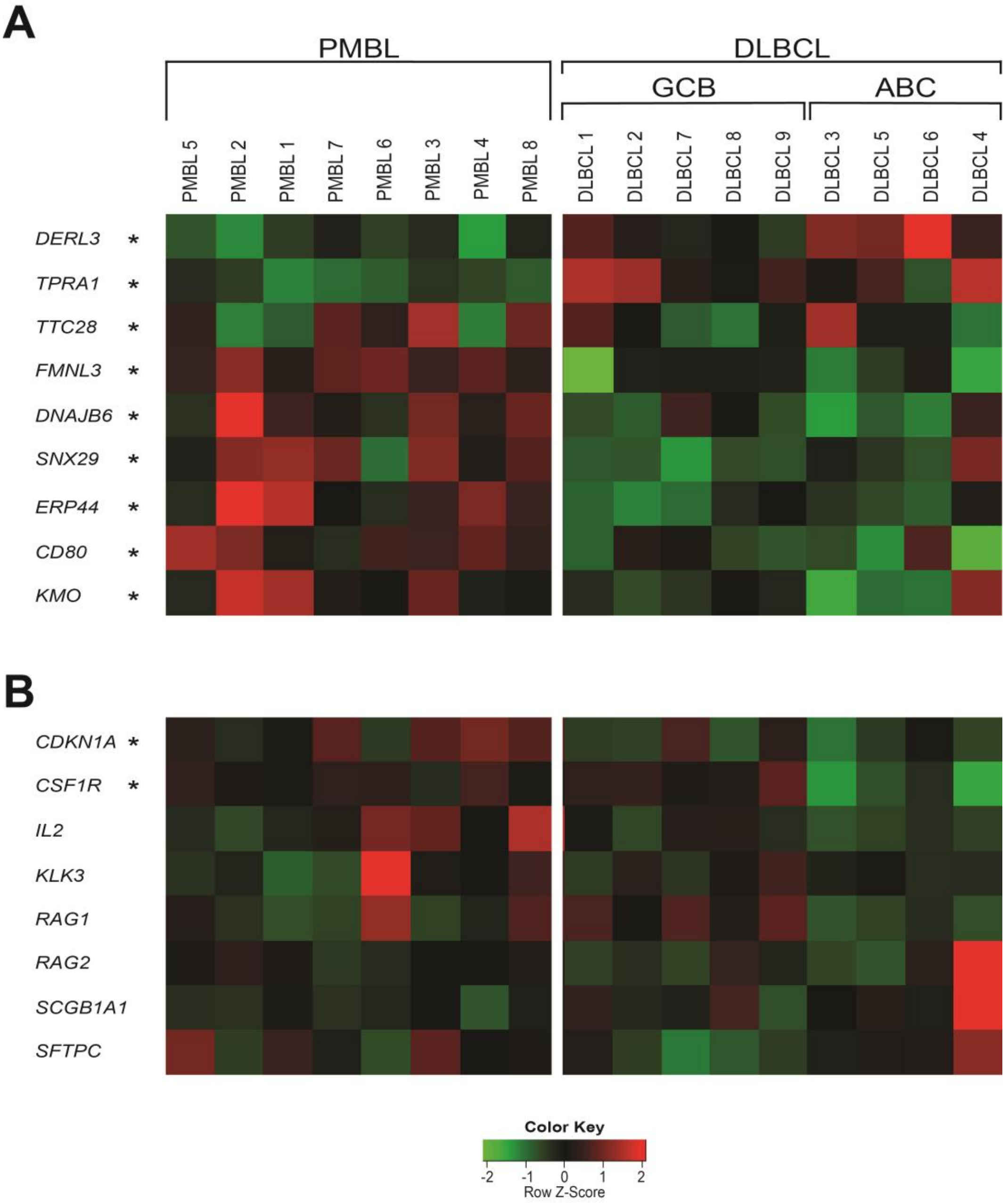

Supplementary figure 7

(A) Comparison of transcriptomic results of PMBL and DLBCL human samples filtered using the FOXP1-linked gene list obtain by Dekker *and al*, [49]. (B) Comparison of transcriptomic results of PMBL and DLBCL human samples filtered using the FOXP1target gene listed in the "transcription factor encyclopedia" database (http://cisreg.cmmt.ubc.ca/). CDKN1A (p21) and CSF1R (M-CSF) are significantly upregulated in PMBL human samples.
